# Trifluridine/tipiracil with and without ramucirumab for advanced gastric cancer: a comparative observational study

**DOI:** 10.1038/s41598-024-61975-7

**Published:** 2024-06-03

**Authors:** Yukiya Narita, Takatsugu Ogata, Yasunobu Ishizuka, Tomoki Sakakida, Munehiro Wakabayashi, Hiroyuki Kodama, Kazunori Honda, Toshiki Masuishi, Hiroya Taniguchi, Shigenori Kadowaki, Masashi Ando, Masahiro Tajika, Kei Muro

**Affiliations:** 1 Department of Clinical Oncology, Aichi Cancer Center Hospital, 1-1 Kanokoden, Chikusa-ku, Nagoya, Aichi 464-8681 Japan; 2 Department of Endoscopy, Aichi Cancer Center Hospital, Nagoya, Japan

**Keywords:** Chemotherapy, Gastric cancer, Ramucirumab, Trifluridine/tipiracil, Cancer, Gastrointestinal diseases, Gastroenterology, Oncology

## Abstract

The combination of trifluridine/tipiracil hydrochloride (FTD/TPI) plus ramucirumab has demonstrated clinical activity in patients with advanced gastric cancer (AGC). We evaluated the efficacy and safety of this combination compared with those of FTD/TPI monotherapy in patients with AGC. We retrospectively reviewed data of patients with AGC who received FTD/TPI plus ramucirumab or FTD/TPI monotherapy as third- or later-line treatment. This study included 36 patients treated with FTD/TPI plus ramucirumab and 70 patients receiving FTD/TPI monotherapy. The objective response rate (ORR) and disease control rate (DCR) were 25.8% and 58.1%, respectively, in the FTD/TPI plus ramucirumab group and 5.0% and 38.3%, respectively, in the FTD/TPI group (ORR, *P* = 0.007; DCR, *P* = 0.081). The median progression-free survival (PFS) was significantly longer in the FTD/TPI plus ramucirumab group (median PFS, 2.9 vs. 1.8 months; hazard ratio [HR]: 0.52; *P* = 0.001). A numerical survival benefit was also observed (median overall survival, 7.9 months vs. 5.0 months; HR: 0.68, *P* = 0.089). In the multivariate analysis, PFS was significantly longer in the FTD/TPI plus ramucirumab group than in the FTD/TPI monotherapy group (HR: 0.61, *P* = 0.030). The incidence of febrile neutropenia was higher in the FTD/TPI plus ramucirumab group than in the FTD/TPI group (13.8% vs. 2.9%); however, no new safety signals were identified. Compared with FTD/TPI monotherapy, FTD/TPI plus ramucirumab offers clinical benefits with acceptable toxicity in heavily pretreated patients with AGC. Further investigation via randomized trials is warranted to confirm these findings.

## Introduction

Gastric cancer is the fifth most common type of cancer and is the fourth-leading cause of cancer-related death^1^. Its incidence and mortality rates are notably high in East Asia. While systemic chemotherapy has prolonged survival of patients with advanced gastric cancer (AGC), median overall survival (OS) remains suboptimal^2^. Several chemotherapeutic agents such as trifluridine/tipiracil hydrochloride (FTD/TPI), irinotecan, nivolumab, and trastuzumab deruxtecan are beneficial as third- or later-line treatment for AGC^3^. Notably, in a large Japanese cohort study of 10,581 patients with AGC receiving palliative systemic chemotherapy, only 2390 patients (22.6%) underwent third-line chemotherapy^4^. This finding highlights the need for further developing treatment strategies in later lines of therapy.

FTD/TPI is an oral medication comprising a nucleoside antitumor component, trifluridine, and a thymidine phosphorylase inhibitor, tipiracil. The TAGS trial of FTD/TPI demonstrated improvement in OS over placebo for heavily pretreated patients with AGC^5^. Preclinical data showed that the combination of FTD/TPI and bevacizumab, a specific angiogenesis inhibitor against vascular endothelial growth factor (VEGF)-A, enhanced antitumor effects compared with FTD/TPI alone^6,7^. Ramucirumab, an anti-VEGF-receptor 2 monoclonal antibody, is an established standard of care for AGC, as evidenced by the results of the REGARD and RAINBOW trials^8,9^. Several single-arm phase II trials of FTD/TPI combined with ramucirumab exhibited promising antitumor activity and feasible safety profile in pretreated patients with AGC^10,11^.

Nivolumab, an immune checkpoint inhibitor targeting programmed cell death-1 (PD-1), has been approved for the primary treatment of AGC and is widely used in clinical practice. Results of a prospective observational study suggested that FTD/TPI monotherapy immediately following nivolumab has a synergistic antitumor effect in AGC, as evidenced by an objective response rate (ORR) of 10.9%^12^. Additionally, the simultaneous inhibition of PD-1 and VEGF pathways can synergize antitumor effects in AGC^13–16^. However, substantial data on the combination therapy of FTD/TPI and ramucirumab following immune checkpoint inhibitors are lacking. Therefore, this retrospective study aimed to evaluate the efficacy and safety of FTD/TPI plus ramucirumab versus FTD/TPI monotherapy in patients with AGC in later-line treatment, most of whom had a history of anti-PD-1 therapy.

## Materials and methods

### Patients

This retrospective study included consecutive patients with AGC who received FTD/TPI plus ramucirumab or FTD/TPI monotherapy as third-line or later treatment at Aichi Cancer Center Hospital between August 2018 and May 2023. The primary inclusion criteria were as follows: (1) histologically confirmed, unresectable, or recurrent gastric cancer; (2) Eastern Cooperative Oncology Group Performance Status (ECOG PS) of 0–2; (3) adequate bone marrow, hepatic, and renal function; (4) ability to maintain adequate oral intake; (5) history of treatment with two or more regimens; and (6) at least one treatment course of FTD/TPI plus ramucirumab or FTD/TPI monotherapy. This study was approved by the Institutional Review Board of Aichi Cancer Center Hospital (No. IR051103) and conformed to the guidelines of the Declaration of Helsinki. The requirement for written informed consent for this study was waived by the Institutional Review Board of Aichi Cancer Center Hospital because of the retrospective, non-interventional design and the available opt-out option on the institution’s website.

### Procedures

FTD/TPI plus ramucirumab therapy involved administering oral FTD/TPI at a dose of 35 mg/m^2^ twice daily on days 1–5 and 8–12 of each 28-day treatment cycle combined with ramucirumab administered intravenously at a dose of 8 mg/kg, repeated every 2 weeks. FTD/TPI monotherapy included oral FTD/TPI at a dose of 35 mg/m^2^ twice daily on days 1–5 and 8–12 of each 28-day cycle. Dose modifications and treatment interruptions were performed at the discretion of each clinician, guided by established clinical trials^5,10^.

### Evaluation of treatment

Clinical data were retrospectively collected from patient medical records. Patients were categorized into FTD/TPI plus ramucirumab and FTD/TPI monotherapy groups for outcome evaluation. Tumor response in patients with measurable lesions was assessed by each clinician according to the Response Evaluation Criteria in Solid Tumors (RECIST) version 1.1^17^. ORR was defined as the proportion of patients with measurable lesions who exhibited either a complete or partial response, as determined by investigators. Disease control rate (DCR) refers to the proportion of patients who achieved complete response, partial response, or stable disease. Progression-free survival (PFS) was calculated from the date of the first administration of the study treatment to the date of disease progression, as indicated by imaging findings, clinical progression, or death owing to any cause. OS was defined from the date of study treatment initiation to the date of death because of any cause or the last follow-up. Adverse events were assessed according to the National Cancer Institute Common Terminology Criteria for Adverse Events version 5.0^18^.

### Statistical analysis

The data cut-off date was November 15, 2023. PFS and OS were estimated using the Kaplan–Meier method, and the stratified log-rank test was utilized to compare variables among patients with respect to survival. Cox proportional hazards regression analysis was employed for survival analysis across different patient groups. The variables included in the multivariate Cox proportional model were selected based on factors with *P-values* < 0.2 in the univariate analysis. Age (≥ 65 vs. < 65 years), sex (male vs. female), ECOG PS (≥ 1 vs. 0), histology (diffuse vs. intestinal), history of gastrectomy (yes vs. no), lymph node metastasis (yes vs. no), liver metastasis (yes vs. no), peritoneal metastasis (yes vs. no), number of metastatic sites (≥ 2 vs. 1), number of prior chemotherapy regimens (≥ 3 vs. 1–2), duration of prior ramucirumab (≥ 3 months vs. < 3 months), ramucirumab-free interval (≥ 3 months vs. < 3 months), and anti-PD-1 inhibitor-free interval (IFI) (< 60 days vs. ≥ 60 days) were incorporated as confounders in the multivariate analysis of PFS and OS. To determine the potential benefits of continuation or re-challenge of ramucirumab and the synergistic effect of anti-PD-1 inhibitor and ramucirumab, cut-off values of three for ramucirumab-free interval and 60 days for IFI were adopted as previously described^19,20^. Exploratory efficacy analyses based on subgroups of liver metastasis (LM) and IFI were conducted, and all outcomes were compared. Statistical analyses were performed using R software version 4.1.0 (R Project for Statistical Computing, Vienna, Austria). All tests were two sided, and *P-values* < 0.05 indicated statistically significant differences.

### Ethics approval

The study was conducted in accordance with the principles of the Declaration of Helsinki and was approved by the Institutional Review Board of Aichi Cancer Center Hospital (No. IR051103).

### Informed consent

The requirement for written informed consent for this study was waived by the Institutional Review Board of Aichi Cancer Center Hospital due to the retrospective study design without intervention, with an opt-out opportunity provided on the institution’s website.

## Results

### Patients

Among 106 patients, 36 and 70 were treated with FTD/TPI plus ramucirumab and FTD/TPI monotherapy, respectively. Table 1 summarizes patient characteristics. The proportions of patients with ECOG PS of 0 (*P* = 0.013) and a ramucirumab-free interval of ≥ 3 months (*P* = 0.028) were significantly higher in the FTD/TPI plus ramucirumab group than in the FTD/TPI monotherapy group. No significant differences in LM and IFI were observed between the two groups. Almost all patients had previously received fluoropyrimidine (100.0% vs. 100.0%), platinum (88.9% vs. 100.0%), taxane (88.9% vs. 100.0%), ramucirumab (86.1% vs. 97.1%), or anti-PD-1 antibody (91.7% vs. 85.7%).

**Table 1:** Patient characteristics.

Characteristics		FTD/TPI		FTD/TPI + ramucirumab		P value
		N = 70	%	N = 36	%	
Age, years	Median (range)	66 (29–85)		65 (38–81)		
	< 65/ ≥ 65	38/32	54.3/45.7	18/18	50.0/50.0	0.687
Sex	Male	43	61.4	22	61.1	1.000
	Female	27	38.6	14	38.9	
ECOG PS	0	17	24.3	18	50.0	0.013
	1	43	61.4	17	47.2	
	2	10	14.3	1	2.8	
Tumor location	EGJ	13	18.6	7	19.4	1.000
	Gastric	57	81.4	29	80.6	
Histology	Diffuse	39	55.7	21	58.3	0.838
	Intestinal	31	44.3	15	41.7	
History of gastrectomy	Yes	28	40.0	15	41.7	1.000
Metastatic site	Lymph node	41	58.6	23	63.9	0.677
	Liver	30	42.9	13	36.1	0.538
	Peritoneum	35	50.0	15	41.7	0.538
	Lung	8	11.4	5	13.9	0.759
Number of metastatic sites	1	21	30.0	12	33.3	0.825
	≥ 2	49	70.0	24	66.7	
Previous chemotherapeutic agent	Fluoropyrimidine	70	100.0	36	100.0	1.000
	Platinum	66	94.3	36	100.0	0.297
	Taxane	70	100.0	32	88.9	0.012
	Irinotecan	15	21.4	11	30.6	0.344
	Ramucirumab	68	97.1	31	86.1	0.043
	Trastuzumab	13	18.6	6	16.7	1.000
	Trastuzumab deruxutecan	10	14.3	4	11.1	0.768
	Anti-PD-1 inhibitor	60	85.7	33	91.7	0.536
Duration of prior ramucirumab^a^	≥ 3 months	48/68	70.6	24/31	77.4	0.628
	< 3 months	20/68	29.4	7/31	22.6	
Treatment pattern of prior ramucirumab^a^	Continue	14/68	20.6	7/31	22.6	0.797
	Re-challenge	54/68	79.4	24/31	77.4	
Ramucirumab-free interval^a^	≥ 3 months	37/68	54.4	24/31	77.4	0.028
	< 3 months	31/68	45.6	7/31	22.6	
Anti-PD-1 inhibitor-free interval^b^	≥ 60 days	26/60	43.3	16/33	48.5	0.668
	< 60 days	34/60	56.7	17/33	51.5	
Number of prior regimens	1–2	9	12.9	7	19.4	0.399
	≥ 3	61	87.1	29	80.6	
HER2	Positive	13	18.6	9	25.0	0.457
	Negative	57	81.4	27	75.0	
MSI status^c^	MSS	40/41	97.6	29/29	100.0	1.000
	MSI-H	1/41	2.4	0/29	0	

^a^The patients who did not recieve ramucirumab before study treatment were excluded from this patient characteristics classification.

^b^The patients who did not recieve anti-PD-1 inhibitor before study treatment were excluded from this patient characteristics classification.

^c^MSI was tested for 41 patients in FTD/TPI group and for 29 patients in FTD/TPI plus ramucirumab group, respectively.

FTD/TPI, trifluridine tipiracil; PD-1, programmed death receptor-1; MSI, microsatellite instability; MSI-H, microsatellite instability high; MSS, microsatellite stable; GEJ, esophagogastric junction; HER2, human epidermal growth factor 2.

### Efficacy outcomes

**Figure 1:**
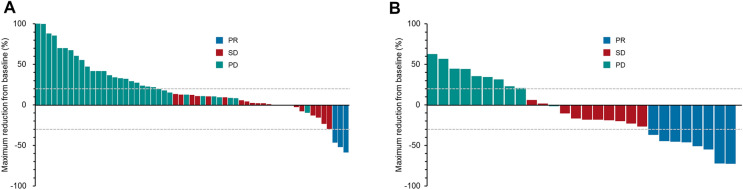
Waterfall plot displaying the percentage changes from baseline in the sum of measurable lesions in the (**A**) FTD/TPI monotherapy and (**B**) FTD/TPI plus ramucirumab groups. FTD/TPI, trifluridine/tipiracil hydrochloride; PR, partial response; SD, stable disease; PD progressive disease.

**Figure 2:**
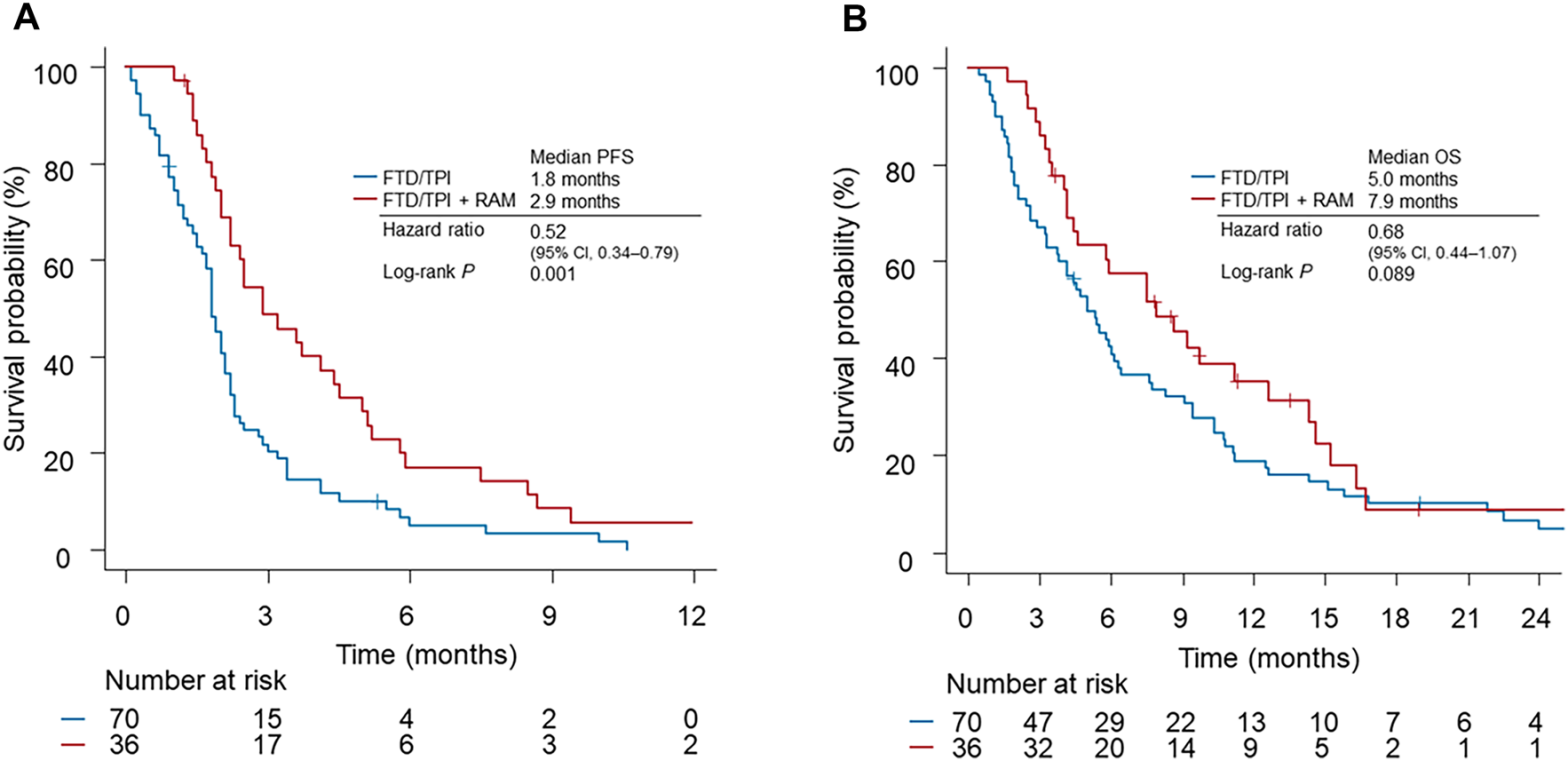
Kaplan–Meier curves of (**A**) progression-free and (**B**) overall survival. FTD/TPI, trifluridine/tipiracil hydrochloride; RAM, ramucirumab; PFS, progression-free survival; OS, overall survival.

At the data cut-off date, the median follow-up time after initiating study treatment was 11.3 months (interquartile range, 8.2–19.0 months). A significantly higher proportion of patients in the FTD/TPI plus ramucirumab group achieved an objective response compared with that in the FTD/TPI monotherapy group (ORR, 25.8% vs. 5.0%, *P* = 0.007) (Table 2, Fig. 1). DCR was numerically better in the FTD/TPI plus ramucirumab group (58.1% vs. 38.3%, *P* = 0.081). Kaplan–Meier curves indicated a significantly longer median PFS in the FTD/TPI plus ramucirumab group (median PFS, 2.9 vs. 1.8 months; hazard ratio [HR]: 0.52, 95% CI 0.34–0.79; *P* = 0.001) (Fig. 2A). A numerical difference in OS favoring the FTD/TPI plus ramucirumab group was also observed (median OS, 7.9 months vs. 5.0 months; HR: 0.68, 95% CI 0.44–1.07; *P* = 0.089) (Fig. 2B). Multivariate analysis showed a significantly longer PFS in the FTD/TPI plus ramucirumab group (HR: 0.61, 95% CI 0.39–0.95; *P* = 0.030) (Table 3) but no significant survival difference between the two groups (HR: 0.97, 95% CI 0.60–1.58; *P* = 0.913) (Table 4).

**Table 2:** Tumor response^a^.

	FTD/TPI		FTD/TPI + ramucirumab		P value
	N = 60	%	N = 31	%	
CR	0	0.0	0	0.0	
PR	3	5.0	8	25.8	
SD	20	33.3	10	32.3	
PD	34	56.7	10	32.3	
NE	3	5.0	3	9.7	
ORR^b^	3	5.0 (95% CI 1.0–13.9)	8	25.8 (95% CI 11.9–44.6)	0.007
DCR^c^	23	38.3 (95% CI 26.1–51.8)	18	58.1 (95% CI 39.1–75.5)	0.081

^a^Tumor response was analyzed for the population with measurable lesions according to RECIST ver. 1.1

^b^ORR was defined as the proportion of patients with CR or PR.

^c^DCR was defined as the proportion of patients with CR or PR or SD.

FTD/TPI, trifluridine tipiracil; RECIST, response evaluation criteria in solid tumors; CR, complete response; PR, partial response; SD, stable disease; PD progressive disease; NE, not evaluable; ORR, objective response rate; DCR, disease control rate; CI, confidence interval.

**Table 3:** Univariate and multivariate analyses for progression-free survival.

Variables	Category	Univariate analysis			Multivariate analysis		
		HR	95% CI	*P*	HR	95% CI	*P*
Age, years	≥ 65 (vs. < 65)	0.92	0.62–1.37	0.687			
Sex	Male (vs. female)	0.77	0.51–1.15	0.203			
ECOG PS	≥ 1 (vs. 0)	1.75	1.14–2.69	0.009	1.59	1.01–2.48	0.044
Histology	Diffuse (vs. intestinal)	1.24	0.83–1.86	0.292			
History of gastrectomy	Yes (vs. no)	1.00	0.66–1.50	0.985			
Lymph node metastasis	Yes (vs. no)	0.90	0.60–1.34	0.589			
Liver metastasis	Yes (vs. no)	0.97	0.64–1.46	0.869			
Peritoneal metastasis	Yes (vs. no)	1.58	1.05–2.36	0.027	1.44	0.94–2.19	0.091
Number of metastatic sites	≥ 2 (vs. 1)	1.34	0.88–2.05	0.173	1.40	0.92–2.14	0.119
Number of prior chemotherapeutic regimens	≥ 3 (vs. 1–2)	0.79	0.45–1.37	0.399			
Duration of prior ramucirumab	≥ 3 months (vs. < 3 months)	0.79	0.52–1.21	0.284			
Ramucirumab-free interval	≥ 3 months (vs. < 3 months)	0.68	0.45–1.04	0.074	0.81	0.52–1.27	0.358
Anti-PD-1 inhibitor-free interval	< 60 days (vs. ≥ 60 days)	0.91	0.61–1.35	0.623			
Chemotherapy	FTD/TPI plus ramucirumab (vs. FTD/TPI)	0.52	0.34–0.79	0.002	0.61	0.39–0.95	0.030

FTD/TPI, trifluridine tipiracil; PD-1, programmed death receptor-1.

**Table 4:** Univariate and multivariate analyses for overall survival.

Variables	Category	Univariate analysis			Multivariate analysis		
		HR	95% CI	*P*	HR	95% CI	*P*
Age, years	≥ 65 (vs. < 65)	1.05	0.70–1.57	0.830			
Sex	Male (vs. female)	0.70	0.46–1.08	0.105	0.84	0.52–1.36	0.475
ECOG PS	≥ 1 (vs. 0)	2.93	1.84–4.67	< 0.001	2.90	1.75–4.79	< 0.001
Histology	Diffuse (vs. intestinal)	1.40	0.93–2.12	0.112	1.05	0.65–1.68	0.848
History of gastrectomy	Yes (vs. no)	1.27	0.84–1.93	0.259			
Lymph node metastasis	Yes (vs. no)	0.94	0.62–1.41	0.763			
Liver metastasis	Yes (vs. no)	0.69	0.45–1.07	0.100	1.03	0.57–1.86	0.925
Peritoneal metastasis	Yes (vs. no)	1.70	1.12–2.57	0.013	1.41	0.80–2.48	0.239
Number of metastatic sites	≥ 2 (vs. 1)	1.15	0.74–1.78	0.530			
Number of prior regimens	≥ 3 (vs. 1–2)	0.78	0.44–1.39	0.405			
Duration of prior ramucirumab	≥ 3 months (vs. < 3 months)	0.74	0.48–1.14	0.165	0.73	0.45–1.16	0.182
Ramucirumab-free interval	≥ 3 months (vs. < 3 months)	0.75	0.49–1.15	0.188	0.80	0.50–1.29	0.358
Anti-PD-1 inhibitor-free interval	< 60 days (vs. ≥ 60 days)	0.84	0.56–1.25	0.391			
Chemotherapy	FTD/TPI plus ramucirumab (vs. FTD/TPI)	0.68	0.44–1.07	0.093	0.97	0.60–1.58	0.913

FTD/TPI, trifluridine tipiracil; PD-1, programmed death receptor-1.

### Outcomes according to LM and IFI

**Figure 3:**
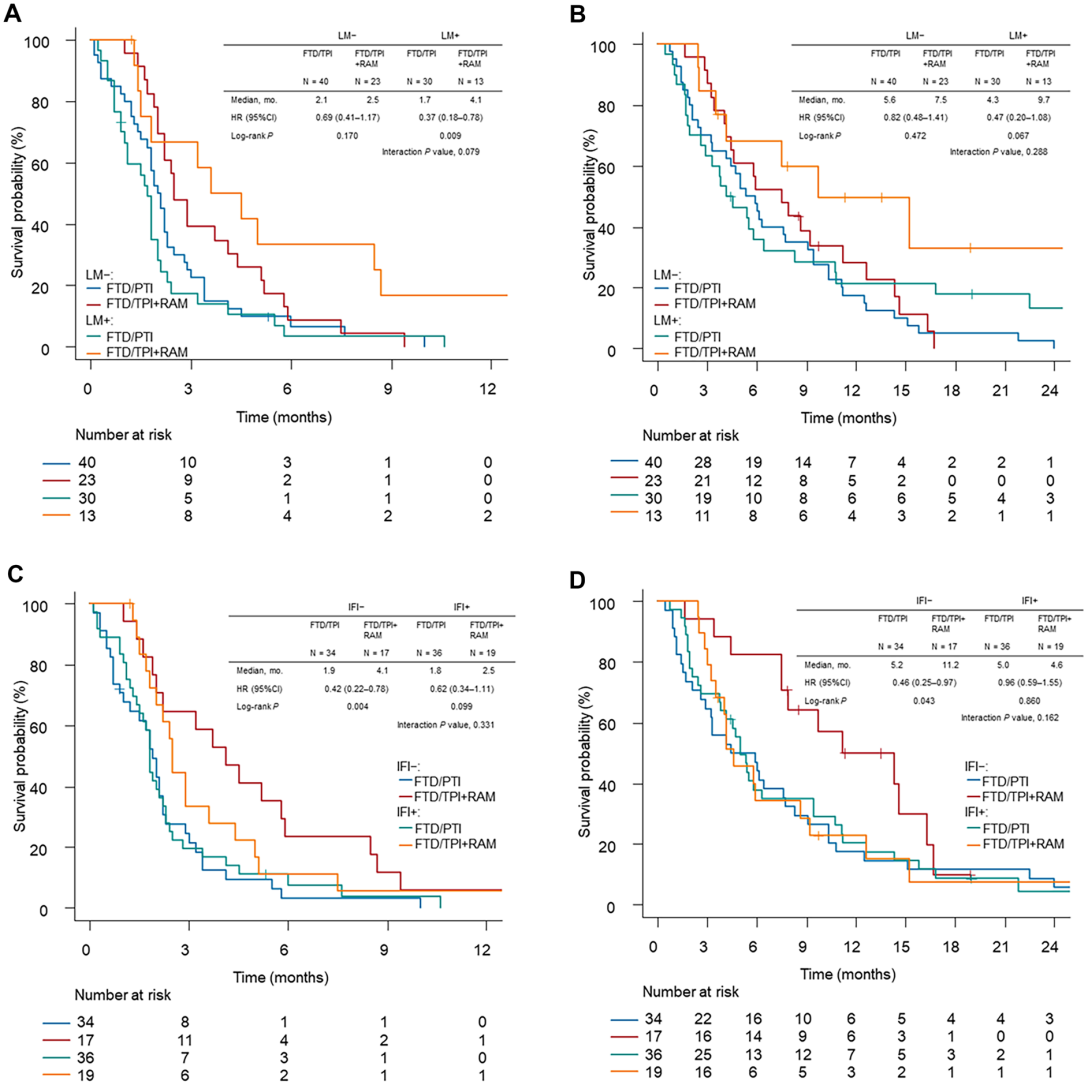
Kaplan–Meier estimates of (**A**) progression-free and (**B**) overall survival according to LM and (**C**) progression-free and (**D**) overall survival according to IFI. FTD/TPI, trifluridine/tipiracil hydrochloride; RAM, ramucirumab; PFS, progression-free survival; OS, overall survival; LM, liver metastasis; IFI, anti-PD-1 inhibitor-free interval; mo., months; HR, hazard ratio; CI, confidence interval.

Kaplan–Meier curves for PFS and OS, stratified by LM and IFI, are shown in Fig. 3. The highest ORR was achieved for LM in the FTD/TPI plus ramucirumab treatment group (ORR, 33.3%) (Supplementary Table 1). Greater treatment benefits on adding ramucirumab were observed in patients with LM (median PFS, 4.1 vs. 1.7 months; HR: 0.37, 95% CI 0.18–0.78; *P* = 0.009) compared with those observed in patients without LM (median PFS, 2.5 vs. 2.1 months; HR: 0.69, 95% CI 0.41–1.17; *P* = 0.170). Similarly, a numerically better survival benefit was observed in patients with LM (median OS, 9.7 vs. 4.3 months; HR: 0.47, 95% CI 0.20–1.08; *P* = 0.067) than in those without LM (median OS, 7.5 vs. 5.6 months; HR: 0.82, 95% CI 0.48–1.41; *P* = 0.472). The interaction *P*-values for PFS (*P* = 0.079) and OS (*P* = 0.288) were not significant. The highest ORR was observed in patients with IFI < 60 days undergoing FTD/TPI plus ramucirumab therapy (ORR, 46.7%) (Supplementary Table 2). The addition of ramucirumab within 60 days of anti-PD-1 inhibitor therapy showed better PFS (median PFS, 4.1 vs. 1.9 months; HR: 0.42, 95% CI 0.22–0.78; *P* = 0.004) and OS (median OS, 11.2 vs. 5.2 months; HR: 0.46, 95% CI 0.25–0.97; *P* = 0.043) than those after 60 days of anti-PD-1 inhibitor therapy (median PFS, 2.5 vs. 1.8 months; HR: 0.62, 95% CI 0.34–1.11; *P* = 0.099: median OS, 4.6 vs. 5.0 months; HR: 0.96, 95% CI 0.59–1.55; *P* = 0.860). The interaction *P*-values for both PFS (*P* = 0.331) and OS (*P* = 0.162) were not significant. No differences in outcomes were observed according to ramucirumab-free interval, duration of prior ramucirumab, and treatment pattern of prior ramucirumab (Supplementary Tables 3–5). The analysis stratified by prior use of immune checkpoint inhibitors showed better PFS in the FTD/TPI monotherapy group with prior use of immune checkpoint inhibitors than without (Supplementary Table 6).

### Safety

Disease progression was the most common reason for discontinuing study treatment in both the groups (91.7% in the FTD/TPI plus ramucirumab group vs. 94.2% in the FTD/TPI monotherapy group), and 2.8% and 2.9% of patients, respectively, discontinued study treatment because of adverse events. After such discontinuation, the proportions of patients receiving subsequent chemotherapy (38.2% vs. 42.6%) and best supportive care (61.8% vs. 57.4%) were similar between the two groups.

Dose reductions of FTD/TPI on initiating study treatment, as determined by each physician, were observed in six patients (16.7%) in the FTD/TPI plus ramucirumab group and 10 patients (14.3%) in the FTD/TPI monotherapy group. During the study treatment, 22 patients (61.1%) required dose reductions because of (N = 12, 33.3%), decrease appetite (N = 6, 16.6%), fatigue (N = 4, 11.1%), nausea (N = 1, 2.7%), and thrombocytopenia (N = 1, 2.7%) in FTD/TPI plus ramucirumab group, whereas 26 patients (37.1%) experienced dosing reductions because of neutropenia (N = 17, 24.2%), decreased appetite (N = 4, 5.7%), fatigue (N = 2, 2.9%), thrombocytopenia (N = 1, 1.4%), nausea (N = 1, 1.4%), and rash (N = 1, 1.4%). No Granulocyte Colony Stimulating Factor preparations as primary prophylaxis were administered in both groups.

Table 5 lists the adverse events occurring during study treatment. The proportion of patients with any grade of decreased appetite was higher in the FTD/TPI plus ramucirumab group than in the FTD/TPI monotherapy group (80.6% vs. 55.7%, *P* = 0.018). Any grade of fatigue was also numerically more common in the FTD/TPI plus ramucirumab group (69.4% vs. 48.6%). Conversely, any grade of anemia was observed numerically less often in the FTD/TPI plus ramucirumab group compared with that in the FTD/TPI monotherapy group (86.1% vs. 98.6%).

**Table 5:** Adverse events.

	FTD/TPI (N = 70)				FTD/TPI plus ramucirumab (N = 36)				*P* value	
	Any grade	%	Grade ≥ 3	%	Any grade	%	Grade ≥ 3	%	Any grade	Grade ≥ 3
Any adverse events	70	100.0	45	64.3	36	100.0	26	72.2	1.000	0.514
Leucopenia	50	71.4	25	35.7	27	75.0	12	33.3	0.819	0.833
Neutropenia	51	72.9	33	47.1	30	83.3	22	61.1	0.334	0.219
Anemia	69	98.6	16	22.9	31	86.1	5	13.9	0.095	0.315
Thrombocytopenia	46	65.7	7	10.0	24	66.7	5	13.9	1.000	0.536
Fatigue	46	65.7	2	2.9	30	83.3	4	11.1	0.070	0.177
Decreased appetite	39	55.7	6	8.6	29	80.6	8	22.2	0.018	0.069
Nausea	34	48.6	2	2.9	25	69.4	1	2.8	0.063	1.000
Vomiting	16	22.9	1	1.4	9	25.0	0	0.0	0.813	1.000
Diarrhea	18	25.7	0	0.0	10	27.8	0	0.0	0.820	1.000
Stomatitis	7	10.0	0	0.0	7	19.4	0	0.0	0.227	1.000
Rash	5	7.1	1	1.4	6	16.7	0	0.0	0.178	1.000
Proteinuria^a^	–	–	–	–	7	19.4	2	5.6	–	–
Febrile neutropenia	–	–	2	2.9	–	–	5	13.8	–	0.043

FTD/TPI, trifluridine tipiracil

^a^The data on proteinuria in the FTD/TPI group was not evaluated.

The incidence of grade 3 or higher adverse events tended to be higher in the FTD/TPI plus ramucirumab group than in the FTD/TPI monotherapy group, although the difference was not statistically significant (72.2% vs. 64.3%, *P* = 0.833). The most common grade 3–4 adverse events were neutropenia (61.1% vs. 47.1%), leukopenia (33.3% vs. 35.7%), decreased appetite (22.2% vs. 8.6%), anemia (13.9% vs. 22.9%), and thrombocytopenia (13.9% vs. 10.0%). The proportion of febrile neutropenia was higher in the FTD/TPI plus ramucirumab group (13.8% vs. 2.9%, *P* = 0.018). No treatment-related deaths were observed in either group.

## Discussion

To the best of our knowledge, this is the largest retrospective study to demonstrate the clinical benefit of FTD/TPI plus ramucirumab over FTD/TPI monotherapy in heavily pretreated patients with AGC. The significant improvements in ORR and PFS in the combination therapy group, though not paralleled by an increase in OS, suggest a notable therapeutic advantage, especially in patients with LM and a short IFI of < 60 days. This observation suggests that certain subgroups of patients with AGC might derive more benefit from this combination therapy.

In the present study, patients in the FTD/TPI plus ramucirumab group showed clinical improvement compared with those in the FTD/TPI monotherapy group. The outcomes of the FTD/TPI monotherapy group (DCR, 38.3%; median OS, 5.0 months) are consistent with those reported in the phase III TAGS trial, suggesting that our cohort is representative of the broader patient population with AGC receiving this treatment (DCR, 44%; median OS, 5.7 months)^5^. Moreover, the addition of ramucirumab in our study (ORR, 25.8%; median PFS, 2.9 months) showed a pattern of clinical benefit comparable with that observed in earlier phase II trials and retrospective studies (ORR, 0–16%; median PFS, 2.9–5.3 months)^10,11,21^. Despite these improvements in ORR and PFS, we did not observe a corresponding increase in OS. This phenomenon, in which improvements in intermediate endpoints do not translate into survival benefits, mirrors findings from other studies, including the RINDBeRG trial, which evaluated the addition of ramucirumab to irinotecan^22^. One possible explanation for the lack of OS benefit in our study could be the higher proportion of patients with favorable baseline characteristics, including ECOG PS of 0 and longer interval without previous ramucirumab exposure, in the combination therapy group. In addition, a substantial proportion of patients (40%) received subsequent chemotherapy. Thus, despite short-term efficacy based on ORR and DCR, these do not translate into a survival benefit. An ongoing randomized phase II trial comparing FTD/TPI plus ramucirumab with FTD/TPI monotherapy will provide further insight into this strategy^23^.

When optimizing ramucirumab treatment for patients with AGC with poor prognosis, identifying clinicopathologic predictors of efficacy is crucial. Our study results showed that adding ramucirumab to FTD/TPI numerically improved PFS and OS in patients with LM, consistent with previous findings that VEGF inhibitors benefit patients with LM across various cancers^24–26^. This result is supported by our previous analysis of 1355 patients with AGC, in which we observed a significant OS improvement in cases with LM post-ramucirumab approval^25^. These results are consistent with those from major trials such as the RAINBOW^25,26^. Preclinical data showed an association between anti-VEGF discontinuation and enhanced liver metastasis, indicating a strong correlation between VEGF and liver metastasis^27^. The mechanism by which VEGF inhibitors are effective against LM remains unclear; however, the unique tumor microenvironment in LM may enhance the effectiveness of VEGF inhibitors^28^.

Our study also explored the impact of prior anti-PD-1 inhibitor use and found only modest improvements in outcomes^12,29^. However, a more pronounced difference was noted in patients with a shorter IFI of < 60 days. One plausible explanation for this difference could be that therapeutic levels of anti-PD-1 inhibitor present at chemotherapy initiation do not persist^30^. No significant differences were observed in outcomes based on the ramucirumab-free interval or previous treatment patterns, consistent with previous efficacy evaluations of VEGF inhibitors^19,22^. This suggests that the timing of anti-PD-1 inhibitor therapy relative to chemotherapy initiation might be crucial.

In our study, a higher proportion of patients in the combination group experienced a decreased appetite (80.6% vs. 55.7%), consistent with data from a phase III trial of FTD/TPI plus a VEGF inhibitor for colorectal cancer^7^. The addition of a VEGF inhibitor to FTD/TPI increased the risk of severe neutropenia without significantly affecting the frequency of febrile neutropenia^7,8,31^. However, we observed that febrile neutropenia was more common in the combination therapy group (13.8%), with a rate higher than those previously reported for AGC treatment but without any treatment-related deaths^10,21^. This increased toxicity trend in the combination therapy group might have influenced treatment choices, especially for patients with high bleeding risks, potentially introducing selection bias. For instance, patients with massive ascites were more likely to receive monotherapy. The occurrence of neutropenia is associated with improved survival outcomes in patients treated with FTD/TPI^32,33^. These findings underscore the critical need to develop effective management strategies, including dose modifications and granulocyte colony-stimulating factor use, tailored to individual patient needs among those receiving FTD/TPI plus ramucirumab therapy.

The retrospective, small sample size, non-randomized, study design is a limitation, potentially introducing selection bias. This is particularly relevant given that treatment decisions were made at the discretion of the treating physicians. Additionally, the fact that a significant proportion of patients in both the groups received subsequent lines of chemotherapy suggests that other factors may have influenced OS.

In conclusion, the study results provide evidence of the clinical benefits of FTD/TPI plus ramucirumab with respect to ORR and PFS, as well as acceptable toxicity, in heavily pretreated patients with AGC. These findings highlight the potential of this combination therapy as a treatment option and underscore the need for further research, ideally through randomized controlled trials, to confirm these results and refine treatment strategies for this patient population.

### Supplementary Information

Supplementary Tables.

## Data Availability

The datasets used and/or analysed during the current study available from the corresponding author on reasonable request.

## Abbreviations

AGC: Advanced gastric cancer
CI: Confidence interval
DCR: Disease control rate
ECOG PS: Eastern Cooperative Oncology Group Performance Status
FTD/TPI: Trifluridine/tipiracil hydrochloride
HR: Hazard ratio
IFI: Anti-PD-1 inhibitor-free interval
LM: Liver metastasis
ORR: Objective response rate
OS: Overall survival
PD-1: Programmed cell death-1
PFS: Progression-free survival
RECIST: Response Evaluation Criteria in Solid Tumors
VEGF: Vascular endothelial growth factor
