# Supplementary material for: Trifluridine/tipiracil with and without ramucirumab for advanced gastric cancer: a comparative observational study
Source: Sci Rep. 2024 Jun 3;14:12658. doi: 10.1038/s41598-024-61975-7 (PMC11148118; doi:10.1038/s41598-024-61975-7)
Supplement: Supplementary file 1 — Supplementary Tables. [file 41598_2024_61975_MOESM1_ESM.docx]

**Supplementary Table 1. Outcomes according to liver metastasis**

|  | Liver metastasis − | | Liver metastasis + | |
| --- | --- | --- | --- | --- |
|  | FTD/TPI  (N = 40) | FTD/TPI + RAM  (N = 23) | FTD/TPI  (N = 30) | FTD/TPI + RAM  (N = 13) |
| Measurable lesion + | 31 | 18 | 29 | 13 |
| ORR^a, b^ | 2 (6.5%) | 6 (33.3%) | 1 (3.4%) | 2 (15.4%) |
| ORR, *P* value | 0.039 | | 0.222 | |
| DCR^a, c^ | 16 (51.6%) | 10 (55.6%) | 7 (24.1%) | 8 (61.5%) |
| DCR, *P* value | 1.000 | | 0.540 | |
| Median PFS (months) | 2.1 | 2.5 | 1.7 | 4.1 |
| PFS, HR^d^ (95% CI) | 0.69 (0.41–1.17) | | 0.37 (0.18–0.78) | |
| PFS, log-rank *P* | 0.170 | | 0.009 | |
| Median OS (months) | 5.6 | 7.5 | 4.3 | 9.7 |
| OS, HR^d^ | 0.82 (0.48–1.41) | | 0.47 (0.20–1.08) | |
| OS, log-rank *P* | 0.472 | | 0.067 | |

^a^: Tumor response was analyzed for the population with measurable lesions according to RECIST ver. 1.1.

^b^: ORR was defined as the proportion of patients with CR or PR.

^c^: DCR was defined as the proportion of patients with CR or PR or SD.

^d^: Liver metastasis (vs. no liver metastasis)

^e^: Less than 60 days of anti-PD-1 inhibitor interval (vs. more than 60 days of anti-PD-1 inhibitor interval)

Abbreviations: RECIST, Response Evaluation Criteria in Solid Tumors; CR, complete response; PR, partial response; SD, stable disease; PD progressive disease; NE, not evaluable; PD-1, programmed death receptor-1; ORR, objective response rate; DCR, disease control rate; CI, confidence interval; PFS, progression-free survival; OS, overall survival; HR, hazard ratio; RAM, ramucirumab

**Supplementary Table 2. Outcomes according to Anti-PD-1 inhibitor-free interval^*^**

|  | Anti-PD-1 inhibitor-free interval < 60 days | | Anti-PD-1 inhibitor-free interval ≥ 60 days | |
| --- | --- | --- | --- | --- |
|  | FTD/TPI  (N = 34) | FTD/TPI + RAM  (N = 17) | FTD/TPI  (N = 36) | FTD/TPI + RAM  (N = 19) |
| Measurable lesion + | 29 | 15 | 31 | 16 |
| ORR^a, b^ | 2 (6.9%) | 7 (46.7%) | 1 (3.2%) | 1 (6.2%) |
| ORR, *P* value | 0.004 | | 1.000 | |
| DCR^a, c^ | 12 (41.4%) | 11 (73.3%) | 11 (35.5%) | 7 (43.8%) |
| DCR, *P* value | 0.060 | | 0.753 | |
| Median PFS (months) | 1.9 | 4.1 | 1.8 | 2.5 |
| PFS, HR^e^ (95% CI) | 0.42 (0.22–0.78) | | 0.62 (0.34–1.11) | |
| PFS, log-rank *P* | 0.004 | | 0.099 | |
| Median OS (months) | 5.2 | 11.2 | 5.0 | 4.6 |
| OS, HR^e^ (95% CI) | 0.46 (0.25–0.97) | | 0.96 (0.59–1.55) | |
| OS, log-rank *P* | 0.043 | | 0.860 | |

^*^: The patients who did not recieve anti-PD-1 inhibitor before study treatment were categorized in the ≥ 60 days group

^a^: Tumor response was analyzed for the population with measurable lesions according to RECIST ver. 1.1.

^b^: ORR was defined as the proportion of patients with CR or PR.

^c^: DCR was defined as the proportion of patients with CR or PR or SD.

^d^: Liver metastasis (vs. no liver metastasis)

^e^: Less than 60 days of anti-PD-1 inhibitor interval (vs. more than 60 days of anti-PD-1 inhibitor interval)

Abbreviations: RECIST, Response Evaluation Criteria in Solid Tumors; CR, complete response; PR, partial response; SD, stable disease; PD progressive disease; NE, not evaluable; PD-1, programmed death receptor-1; ORR, objective response rate; DCR, disease control rate; CI, confidence interval; PFS, progression-free survival; OS, overall survival; HR, hazard ratio; RAM, ramucirumab

**Supplementary Table 3. Outcomes according to** **ramucirumab-free interval**^*^

|  | FTD/TPI | | FTD/TPI plus ramucirumab | | |
| --- | --- | --- | --- | --- | --- |
|  | Ramucirumab-free interval | | | | |
|  | ≥ 3 months  (N = 39) | < 3 months  (N = 31) | | ≥ 3 months  (N = 29) | < 3 months  (N = 7) |
| Measurable lesion + | 34 | 26 | | 26 | 5 |
| ORR^a, b^ | 1 (2.9%) | 2 (7.7%) | | 8 (30.8%) | 0 (0.0%) |
| DCR^a, c^ | 16 (47.1) | 7 (26.9%) | | 16 (61.5%) | 2 (40.0%) |
| Median PFS (months) | 2.0 | 1.8 | | 3.2 | 2.9 |
| PFS, HR | 0.75 | | | 0.99 | |
| PFS, log-rank *P* | 0.234 | | | 0.986 | |
| Median OS (months) | 5.3 | 4.5 | | 8.6 | 7.5 |
| OS, HR | 0.76 | | | 1.30 | |
| OS, log-rank *P* | 0.269 | | | 0.629 | |

^*^: The patients who did not recieve ramucirumab before study treatment were categorized in the ≥ 3 months group

^a^: Tumor response was analyzed for the population with measurable lesions according to RECIST ver. 1.1.

^b^: ORR was defined as the proportion of patients with CR or PR.

^c^: DCR was defined as the proportion of patients with CR or PR or SD.

Abbreviations: RECIST, Response Evaluation Criteria in Solid Tumors; CR, complete response; PR, partial response; SD, stable disease; PD progressive disease; NE, not evaluable; ORR, objective response rate; DCR, disease control rate; PFS, progression-free survival; OS, overall survival; HR, hazard ratio

**Supplementary Table 4. Outcomes according to** **duration of prior ramucirumab**^*^

|  | FTD/TPI | | FTD/TPI plus ramucirumab | | |
| --- | --- | --- | --- | --- | --- |
|  | Duration of prior ramucirumab | | | | |
|  | ≥ 3 months  (N = 48) | < 3 months  (N = 22) | | ≥ 3 months  (N = 25) | < 3 months  (N = 11) |
| Measurable lesion + | 41 | 19 | | 22 | 9 |
| ORR^a, b^ | 3 (7.3%) | 0 (0.0%) | | 6 (27.3%) | 2 (22.2%) |
| DCR^a, c^ | 15 (36.6%) | 8 (42.1%) | | 12 (54.5%) | 6 (66.7%) |
| Median PFS (months) | 1.90 | 1.75 | | 3.4 | 2.5 |
| PFS, HR | 0.84 | | | 0.78 | |
| PFS, log-rank *P* | 0.492 | | | 0.502 | |
| Median OS (months) | 5.0 | 5.0 | | 7.9 | 9.2 |
| OS, HR | 0.71 | | | 0.80 | |
| OS, log-rank *P* | 0.196 | | | 0.595 | |

^*^: The patients who did not recieve ramucirumab before study treatment were categorized in the < 3 months group

^a^: Tumor response was analyzed for the population with measurable lesions according to RECIST ver. 1.1.

^b^: ORR was defined as the proportion of patients with CR or PR.

^c^: DCR was defined as the proportion of patients with CR or PR or SD.

Abbreviations: RECIST, Response Evaluation Criteria in Solid Tumors; CR, complete response; PR, partial response; SD, stable disease; PD progressive disease; NE, not evaluable; ORR, objective response rate; DCR, disease control rate; PFS, progression-free survival; OS, overall survival; HR, hazard ratio

**Supplementary Table 5. Outcomes according to** **treatment pattern of prior ramucirumab**^*^

|  | FTD/TPI | | FTD/TPI plus ramucirumab | | |
| --- | --- | --- | --- | --- | --- |
|  | Treatment pattern of prior ramucirumab | | | | |
|  | Continue  (N = 14) | Re-challenge  (N = 56) | | Continue  (N = 7) | Re-challenge  (N = 29) |
| Measurable lesion + | 11 | 49 | | 5 | 26 |
| ORR^a, b^ | 1 (9.1%) | 2 (4.1%) | | 1 (20.0%) | 7 (26.9%) |
| DCR^a, c^ | 4 (36.4%) | 7 (38.8%) | | 2 (40.0%) | 16 (61.5%) |
| Median PFS (months) | 1.8 | 1.9 | | 2.9 | 3.2 |
| PFS, HR | 0.84 | | | 0.73 | |
| PFS, log-rank *P* | 0.563 | | | 0.487 | |
| Median OS (months) | 5.8 | 5.0 | | 7.5 | 8.6 |
| OS, HR | 0.97 | | | 0.96 | |
| OS, log-rank *P* | 0.928 | | | 0.943 | |

^*^: The patients who did not recieve ramucirumab before study treatment were categorized in the re-challenge group

^a^: Tumor response was analyzed for the population with measurable lesions according to RECIST ver. 1.1.

^b^: ORR was defined as the proportion of patients with CR or PR.

^c^: DCR was defined as the proportion of patients with CR or PR or SD.

Abbreviations: RECIST, Response Evaluation Criteria in Solid Tumors; CR, complete response; PR, partial response; SD, stable disease; PD progressive disease; NE, not evaluable; ORR, objective response rate; DCR, disease control rate; PFS, progression-free survival; OS, overall survival; HR, hazard ratio

**Supplementary Table 6. Outcomes according to history of** **prior use of immune checkpoint inhibitor**

|  | FTD/TPI | | FTD/TPI plus ramucirumab | | |
| --- | --- | --- | --- | --- | --- |
|  | Prior use of immune checkpoint inhibitor | | | | |
|  | Yes  (N = 60) | No  (N = 10) | | Yes  (N = 33) | No  (N = 3) |
| Measurable lesion + | 52 | 8 | | 29 | 2 |
| ORR^a, b^ | 3 (5.8%) | 0 (0.0%) | | 8 (27.6%) | 0 (0.0%) |
| DCR^a, c^ | 20 (38.5%) | 3 (37.5%) | | 17 (58.6%) | 1 (50.0%) |
| Median PFS (months) | 1.9 | 1.4 | | 2.9 | 5.0 |
| PFS, HR | 0.45 | | | 1.04 | |
| PFS, log-rank *P* | 0.02 | | | 0.943 | |
| Median OS (months) | 5.5 | 3.8 | | 7.9 | 12.6 |
| OS, HR | 0.64 | | | 0.93 | |
| OS, log-rank *P* | 0.19 | | | 0.912 | |

^a^: Tumor response was analyzed for the population with measurable lesions according to RECIST ver. 1.1.

^b^: ORR was defined as the proportion of patients with CR or PR.

^c^: DCR was defined as the proportion of patients with CR or PR or SD.

Abbreviations: RECIST, Response Evaluation Criteria in Solid Tumors; CR, complete response; PR, partial response; SD, stable disease; PD progressive disease; NE, not evaluable; ORR, objective response rate; DCR, disease control rate; CI, confidence interval; PFS, progression-free survival; OS, overall survival; HR, hazard ratio
